# Exploring the Role of Social Workers in Age Friendly Health Systems: A Scoping Review

**DOI:** 10.1093/geroni/igaf122.526

**Published:** 2025-12-31

**Authors:** Leslie Hasche, Aprille Arena, Jina Park

**Affiliations:** University of Denver, Denver, Colorado, United States; University of Denver, Denver, Colorado, United States; University of Denver, Denver, Colorado, United States

## Abstract

Age-friendly health systems have expanded across academic medicine, hospital systems, Center for Medicare and Medicaid payment mechanisms, and federal geriatric workforce efforts. Age friendly health systems involve a 4M Framework of (1) What Matters, (2) Medication, (3) Mentation, and (4) Mobility, with corresponding implementation tools, quality measures, and interprofessional training programs. Given that 30% of social workers are employed in hospital, health care, or long-term care settings, the specific role of social workers warrants exploration. This scoping review aimed to identify how social workers, or social services have engaged in age-friendly health care. The search, conducted in March of 2025, originally yielded 159 articles. Following duplication removal and eligibility screening, 24 articles were included. Over half of the articles were commentary or review articles describing age-friendly health systems that incorporated naming social work as a key interprofessional role or explicated how social workers may be involved in geriatric emergency room responses, assessments, caregiver support, and care management. Empirical articles included eight qualitative or mixed method studies that (1) evaluated implementation or training efforts, (2) identified unmet needs, or (3) described barriers. As part of the mixed methods studies and in two unique quantitative studies, retrospective analysis of clinical chart data was used to assess health outcomes. Social workers did offer unique contributions to promote consideration of disability-friendly care needs and of cultural adaptations, particularly for older Hawaiian populations. Geriatric social work education efforts should be updated to incorporate age-friendly health care approaches and attend to adaptations for diverse older adults.

